# Comparative real-world outcomes of stage III melanoma patients treated with talimogene laherparepvec or interleukin 2

**DOI:** 10.1177/17588359251324035

**Published:** 2025-04-01

**Authors:** Markus Reitmajer, Lena Nanz, Nina Müller, Ulrike Leiter, Teresa Amaral, Valentin Aebischer, Lukas Flatz, Andrea Forschner

**Affiliations:** Department of Dermatology, University Hospital Tuebingen, Liebermeisterstraße 25, Tuebingen 72076, Germany; Department of Dermatology, University Hospital Tuebingen, Tuebingen, Germany; University Pharmacy, University Hospital Tuebingen, Tuebingen, Germany; Department of Dermatology, University Hospital Tuebingen, Tuebingen, Germany; Department of Dermatology, University Hospital Tuebingen, Tuebingen, Germany; Department of Dermatology, University Hospital Tuebingen, Tuebingen, Germany; Department of Dermatology, University Hospital Tuebingen, Tuebingen, Germany; Department of Dermatology, University Hospital Tuebingen, Tuebingen, Germany

**Keywords:** cutaneous metastases, interleukin 2 (IL-2), intralesional treatment, melanoma, overall survival (OS), progression-free survival (PFS), skin metastases, talimogene laherparepvec (T-VEC)

## Abstract

**Background:**

Talimogene laherparepvec (T-VEC) and interleukin-2 (IL-2) are both used in the intralesional treatment of melanoma skin metastases. T-VEC received regulatory approval from the European Medicines Agency and the U.S. Food and Drug Administration in 2015, while IL-2 has been used off-label for this purpose for many years. Despite their use in clinical practice, there is a lack of comparative data on the efficacy and safety of these treatments.

**Objectives:**

This retrospective study aimed to compare the efficacy and safety of intralesional T-VEC and IL-2 in non-resectable stage III patients with melanoma treated at a single center between January 2016 and September 2024.

**Methods:**

We identified eligible patients using the Central Malignant Melanoma Registry and the local University Hospital Pharmacy database. Overall survival (OS) and progression-free survival (PFS) were calculated. Furthermore, best response rates and occurrence of adverse events (AEs) were compared between the T-VEC and the IL-2 group. Concomitant systemic treatment was allowed.

**Results:**

A total of 62 patients were included, with 37 receiving T-VEC and 25 receiving IL-2 as first-line therapy. Ten patients received both therapies subsequently. The median PFS for the cohort was 5.0 months, and the median OS was 34.0 months. No significant differences in PFS (*p* = 0.790), OS (*p* = 0.894), or best response rates (*p* = 0.468) were found between groups. Common AEs included local injection site reactions and fever, with no severe events leading to discontinuation by a physician.

**Conclusion:**

No significant differences in PFS, OS, or best response rates were observed between IL-2 and T-VEC treatments. The choice of therapy may be influenced by factors such as availability, physician preference, and patient-specific considerations.

## Plain language summary

Study comparing real-world survival and treatment response in stage III melanoma patients using talimogene laherparepvec or interleukin 2

Why was the study done?

Talimogene laherparepvec (T-VEC) and interleukin-2 (IL-2) are both used for the intralesional treatment of melanoma skin metastases. T-VEC received regulatory approval from the European Medicines Agency and the U.S. Food and Drug Administration in 2015. In contrast, IL-2 has been used off-label for this purpose for many years but lacks official approval. Despite their concurrent use in clinical practice, there is limited data directly comparing the efficacy and safety of these treatments.

What did the researchers do?

This retrospective study compared the efficacy and safety of intralesional T-VEC and IL-2 in patients with non-resectable stage III melanoma. The patients were treated at a single center between January 2016 and September 2024. Researchers identified eligible patients using data from the Central Malignant Melanoma Registry and the local University Hospital Pharmacy database. They calculated overall survival (OS) and progression-free survival (PFS) for these patients. Additionally, the study compared the best response rates and the occurrence of adverse events between the T-VEC and IL-2 groups.

What did the researchers find?

A total of 62 patients were included in the study: 37 received intralesional T-VEC treatment, and 25 received IL-2 treatment as first-line therapy. Additionally, 10 patients received both therapies sequentially. The median progression-free survival (PFS) for the cohort was 5.0 months, and the median overall survival (OS) was 34.0 months. No significant differences were observed between the two groups in terms of PFS (*p* = 0.790), OS (*p* = 0.894), or best response rates (*p* = 0.468). Common adverse events included local injection site reactions and fever. No severe adverse events were observed, and consequently, no treatments had to be discontinued by a physician.

What do the findings mean?

No significant differences in PFS, OS or best response rates were observed between IL-2 and T-VEC treatments.

## Introduction

In addition to systemic immune checkpoint inhibitors (ICI) and targeted therapies with BRAF and MEK inhibitors, locally applied intralesional therapies play an important role in the treatment of melanoma skin metastases. Due to their visibility, skin metastases can cause psycho-oncological distress and impair quality of life. In addition, they may lead to physical symptoms, such as ulceration, bleeding, or unpleasant odor.^1,2^ The genetically engineered oncolytic viral therapy, talimogene laherparepvec (T-VEC), has been approved by both the European Medicines Agency (EMA) and the U.S. Food and Drug Administration (FDA) since 2015 for the treatment of unresectable melanoma metastases in the skin without metastatic involvement of distant organs.^3–9^ T-VEC induces anti-tumor effects in two important ways: direct local tumor lysis and indirect effect by inducing a systemic antigen-specific T-cell response against the malignant tumor cells.^8,10^ T-VEC requires strict biosafety protocols and logistical precautions due to the risk of viral transmission. It is classified as a biosafety level 1 agent.^11,12^

Furthermore, intratumorally injected interleukin 2 (IL-2) and IL-2-containing agents have shown promising response rates and have been used to treat melanoma skin metastases for years.^13–15^ However, the number of publications reporting data on patients treated with local IL-2 therapy is limited. Nevertheless, the little data available revealed impressive results with response rates of more than 80%.^16–19^ In a recently published retrospective single-center study of 27 patients, the addition of intralesional IL-2 to systemic ICI therapy was associated with improved progression-free survival (PFS) and overall survival (OS) in the subgroup of patients with locoregional progression and no distant metastases.^20^ However, the combination of T-VEC and pembrolizumab did not improve PFS or OS compared to T-VEC-placebo plus pembrolizumab in a randomized, double-blind, placebo-controlled phase III trial (Masterkey-265) involving 692 patients with advanced melanoma.^21^

Although both T-VEC and IL-2 have been used for years, no prospective or retrospective direct comparative data are available regarding response rates, survival outcomes, or safety profiles. In this retrospective study, we aimed to investigate whether there are differences in PFS and OS rates or safety profiles between T-VEC and intralesional IL-2 in a cohort of patients with non-resectable stage III melanoma.

## Methods

### Study design

We used the institutional database from the Central Malignant Melanoma Registry (CMMR) and the local University Hospital Pharmacy to identify potentially eligible patients with cutaneous metastases who received intralesional treatments with T-VEC and/or IL-2, between January 2016 and September 2024. All patients who received either T-VEC and/or IL-2 treatment were included to prevent selection bias. Concomitant systemic treatment was allowed. In the next step, we collected data on adverse events (AEs), concomitant systemic therapies, local treatment start and end dates, age, gender, histological type, and other clinical characteristics from the available medical records (Tables 1 and 2). All patients with stage III melanoma baseline at the start of intralesional therapy were included. If patients had subsequently both treatments, IL-2 and T-VEC, the first one was considered, and all subsequent therapies were reported separately. The primary endpoint was PFS. The secondary endpoint was OS. OS was defined as the time between the start of intralesional treatment and death or last contact date if the patient was alive. PFS was defined as the time between the start of intralesional treatment and progression and death or last contact date if the patient was alive. Apart from this, we evaluated the documented local best response and AEs between the two intralesional therapies. All therapeutic decisions were made by an interdisciplinary tumor board, and complete R0 resection was recommended instead of intralesional therapy whenever it was considered feasible. The reporting of this study conforms to the STROBE statement.^22^ All patients included in the CMMR provided written informed consent for documentation of their clinical data for research purposes and publications. This study complies with the Declaration of Helsinki and was approved by the local Ethics Committee of the University of Tuebingen (project number 040/2024BO2).

**Table 1.** Clinical and histopathologic characteristics.

Clinical and histopathologic characteristics	Total cohort		T-VEC		IL-2		*p* ^C/M^
	*N*	%	*N*	%	*N*	%	
Sex							0.498^C^
Female	33	53.2	21	56.8	12	48.0	
Male	29	46.8	16	43.2	13	52.0	
Age at time of first diagnosis—median (range)	72.0 (37–93)		70.0 (43–88)		73.0 (37–93)		0.518^M^
Age at time of start intralesional therapy—median (range)	75.0 (42–94)		75.0 (46–90)		75.0 (42–94)		0.852^M^
Age at the time of stage III diagnosis—median (range)	73.0 (41–93)		73.0 (44–88)		74.0 (41–93)		0.566^M^
Stage at primary diagnosis							0.114^C^
IA	2	3.2	0	0	2	8.0	
IB	5	8.1	4	10.8	1	4.0	
IIA	8	12.9	3	8.1	5	20.0	
IIB	3	4.8	1	2.7	2	8.0	
IIC	7	11.3	5	13.5	2	8.0	
IIIA	12	19.4	10	27.0	2	8.0	
IIIB	13	21.0	9	24.3	4	16.0	
IIIC	12	19.4	5	13.5	7	28.0	
Stage at start intralesional therapy							0.626^C^
IIIB	14	22.6	8	21.6	6	24.0	
IIIC	43	69.4	25	67.6	18	72.0	
IIID	5	8.1	4	10.8	1	4.0	
Melanoma type							0.649^C^
Superficial spreading melanoma	19	30.6	11	29.7	8	32.0	
Nodular melanoma	14	22.6	9	24.3	5	20.0	
Acral lentiginous melanoma	19	30.6	13	35.1	6	24.0	
Lentigo maligna melanoma	3	4.8	1	2.7	2	8.0	
Occult melanoma	1	1.6	0	0	1	4.0	
No data available	6	9.7	3	8.1	3	12.0	
Ulceration							0.615^C^
Yes	30	48.4	16	43.2	14	56.0	
No	29	46.8	19	51.4	10	40.0	
No data available	3	4.8	2	5.4	1	4.0	

The stage at primary diagnosis and the start of therapy was determined according to the AJCC classification from 2017. Chi-square test (^C^), median test (^M^).

AJCC, American Joint Committee on Cancer; IL-2, interleukin 2; *N*, number of patients; *p, p*-value; T-VEC, talimogene laherparepvec.

**Table 2.** Intralesional treatment-related characteristics.

Treatment-related characteristics	Total cohort						
	*N*		% of the total cohort				
Patients with melanoma stage III who started an intralesional therapy between 01/2016 and 09/2024	62		100				
Treatment with T-VEC	37		59.7				
Treatment with IL-2	25		40.3				
Treatment with both intralesional therapies	10		16.1				
First T-VEC, followed by IL-2	7		11.3				
First IL-2, followed by T-VEC	3		4.8				
	Total cohort		T-VEC		IL-2		***p*** ^C/L/M^
	***N***	% of the total cohort	***N***	% of category	***N***	% of category	
Entry into stage IV diagnosis							0.502^C^
Yes	28	45.2	18	48.6	10	40.0	
No	34	54.8	19	51.4	15	60.0	
Local best response to intralesional therapy							
ORR	23	37.1	12	32.4	11	44.0	0.355^C^
CR	15	24.2	9	24.3	6	24.0	0.468^F^
PR	8	12.9	3	8.1	5	20.0	
SD	7	11.3	6	16.2	1	4.0	
PD	28	45.2	17	45.9	11	44.0	
n/a	4	6.5	2	5.4	2	8.0	
Alive at the time of evaluation (09/2024)							0.570^C^
Yes	32	51.6	18	48.6	14	56.0	
No	30	48.4	19	54.4	11	44.0	
Duration of intralesional therapy in weeks at the time of evaluation (09/2024)—median (range)	10.0 (1–99)		12.0 (1–99)		6.0 (1–50)		0.024^M^
Still receiving intralesional therapy at the time of evaluation (09/2024)							0.144^C^
Yes	4	6.5	1	2.7	3	12.0	
No	58	93.5	36	97.3	22	88.0	
Median follow-up time after the initiation of intralesional therapy (range)	20 (1–86)		22 (1–80)		7 (1–86)		0.315^M^
	Total cohort		T-VEC		IL-2		***p*** ^C/L/M^
	***N***	% of the total cohort	***N***	% of category	***N***	% of category	
Adverse events associated with the applied intralesional therapies							0.141^C^
Yes	44	71.0	23	62.2	21	84.0	
No	16	25.8	12	32.4	4	16.0	
No data available	2	3.2	2	5.4	0	0	
Concomitant systemic therapies							0.787^C^
Yes	34	54.8	21	56.8	13	52.0	
No	28	45.2	16	43.2	12	48.0	
Type of concomitant systemic therapy							0.683^C^*
ICI	33	53.2	20	54.1	13	52.0	
TT	1	1.6	1	2.7	0	0	
Timing of concomitant systemic therapy							0.647^F^
Started before intralesional therapy	23	67.6	15	71.4	8	61.5	
Started together with intralesional therapy	10	29.4	6	28.6	4	30.8	
Started later due to regional lymph node progression	1	2.9	0	0	1	7.7	
Median PFS in months (95% CI)	5 (0.01–11.80)		5 (2.51–7.49)		5 (0.01–10.80)		0.790^L^
Median OS in months (95% CI)	34 (23.35–44.65)		31.0 (12.65–49.35)		40.0 (18.76–61.24)		0.894^L^

No data are available due to death before the first evaluation or failure to attend the appointment (n/a). Chi-square test (^C^), median test (^M^), log-rank test (^L^), and Fisher’s exact test (^F^).

CI, confidence interval; CR, complete response; ICI, immune checkpoint inhibitors; *N*, number of patients; ORR, overall response rate; OS, overall survival; *p, p*-value; PD, progressive disease; PFS, progression-free survival; PR, partial response; SD, stable disease; TT, targeted therapies; T-VEC, talimogene laherparepvec.

### Application of the intralesional therapies and follow-up

IL-2 was scheduled according to the following scheme: application over 5 days with increasing dosages, starting with 3 million IU on the first day, followed by 6 million IU on the second day, and 9 million IU on days 3–5. The patient was required to remain hospitalized for five consecutive nights during the course of therapy. T-VEC was applied according to the company’s instructions. In the first cycle, the maximum total injection volume is up to 4 ml, with a concentration of 10^6^ PFU/ml. In the second cycle, 3 weeks later, the maximum total injection volume is up to 4 ml, with a concentration of 10^8^ PFU/ml. All following cycles, including any potential re-inductions, are scheduled 2 weeks after the previous cycle. The maximum total injection volume is always up to 4 ml, with a concentration of 10^8^ PFU/ml. The T-VEC applications were carried out in an outpatient setting. In both intralesional therapies, dose escalation and continuation of therapy were performed according to tolerability and patient consent. The staging was performed by whole-body imaging (positron emission tomography-computed tomography (PET-CT), CT, or magnetic resonance imaging) complemented by locoregional sonography (US) of the primary tumor scar, in-transit route and lymph nodes, and photo-documentation if ultrasound was insufficient to assess the tumor extends, that is, very small and superficial lesions. In case of uncertainty regarding the presence of vital tumor cells, punch biopsies (4 mm) were taken and histopathological evaluation was performed at the local Institute of Dermatopathology at the University Hospital Tuebingen, Germany.

### Statistical analysis

Demographic and clinical data were characterized using statistical descriptive analyses conducted with IBM^®^ SPSS^®^ Statistics 28.0 (IBM, Armonk, NY, USA), STATA version 15.1 (StataCorp LLC, College Station, TX, USA), and GraphPad PRISM^®^ 9.5.0 (Dotmatics, Boston, MA, USA). The chi-square test was used to compare clinical, histopathologic, and treatment-associated characteristics. When the expected cell frequencies were below 5, a Fisher’s exact test was performed. A median test (Mann–Whitney *U* test) was performed to assess differences in the medians among the two groups (details in Tables 1 and 2). Survival analyses were conducted using the Kaplan–Meier method. Log-rank test was used to compare survival distributions. Throughout the analysis, *p*-values of less than 0.05 were considered statistically significant. Graphs were generated using Office PowerPoint^®^ 2019 (Microsoft, Redmond, Washington, USA), STATA version 15.1 (StataCorp LLC), and GraphPad PRISM 9.5.0 (Dotmatics, USA).

## Results

### Characterization of the cohort

**Figure 1.**
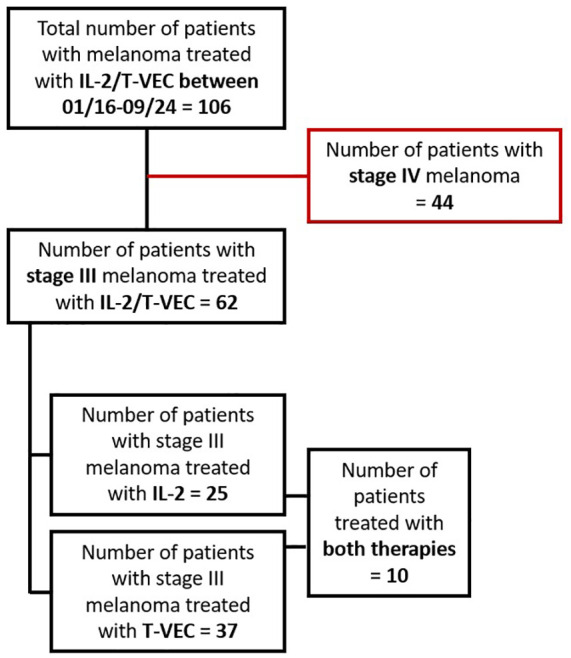
Flowchart of patients included in the analysis.

From January 2016 to September 2024, a total of 106 patients were treated with intralesional therapies at the University Hospital in Tuebingen. Forty-four patients had simultaneous distant metastases (stage IV) at the timepoint of treatment initiation of intralesional therapy and were therefore excluded from this analysis. A total of 62 patients with stage III melanoma were included in this retrospective study. Of these 62 patients, 37 were injected with T-VEC, and 25 were treated with IL-2. Ten patients received both intralesional therapies during the course of their disease (Figure 1). Seven patients received T-VEC first, followed by IL-2, while three patients received IL-2 first, followed by T-VEC (Supplemental Table 1). The most recently enrolled patient began intralesional therapy on May 13, 2024. The median follow-up time after the initiation of intralesional therapy was 20 months (range: 1–86) with no significant difference between the groups (*p* = 0.315; Table 2).

Among the cohort of 62 patients treated with intralesional therapies, 53.2% were female and 46.8% were male. The median age at diagnosis was 72.0 years (range: 37.0–93.0), the median age at the start of the intralesional therapy was 75.0 years (range: 42–94), and the median age at the time of stage III diagnosis was 73.0 years (range: 41.0–93.0). The distribution of the stage at the start of intralesional therapies was as follows: 69.4% with IIIC, 22.6% with IIIB, and 8.1% with IIID. With 30.6% each, the superficial spreading and acral lentiginous histological subtypes were the two most common histological subtypes included in the cohort. These were followed by nodular melanoma at 22.6%, lentigo maligna melanoma at 4.8%, and occult melanoma at 1.6%. In six patients (9.7%), no information regarding the histological subtype was found in the available medical records. Ulceration was present in 48.4% of the primary lesions (Table 1).

More than half of the patients (54.8%) received concomitant systemic therapy, with the majority receiving ICI therapy (53.2% of the cohort; 97.1% of the patients with concomitant systemic therapy). In both subgroups (IL-2 and T-VEC), the most common situation, observed in more than two-thirds of cases, was that concomitant systemic therapy was initiated in an adjuvant setting prior to the start of intralesional therapy and continued thereafter. In only one case, in the IL-2 subgroup, was the patient resected with an R1 status, so the concomitant systemic therapy was not adjuvant. In approximately 30% of cases, the concomitant systemic therapy was started at the same time as the intralesional therapy. In the IL-2 subgroup, one patient received additional concomitant systemic therapy during intralesional treatment following the development of a regional lymph node metastasis. However, no patients in either subgroup started additional concomitant systemic therapy later due to distant disease progression. No significant difference regarding the timing or situation of concomitant therapy was observed between the two groups (*p* = 0.647). The two subgroups (IL-2 and T-VEC) did not differ significantly in terms of median age, age at the time of start of intralesional therapy, gender, histological subtype, ulceration, stage at primary diagnosis/start of intralesional therapy, concomitant systemic therapy, or other clinical, histopathological, and treatment-associated baseline characteristics (Tables 1 and 2).

### Similar survival outcomes and best response among the intralesional therapies

**Figure 2.**
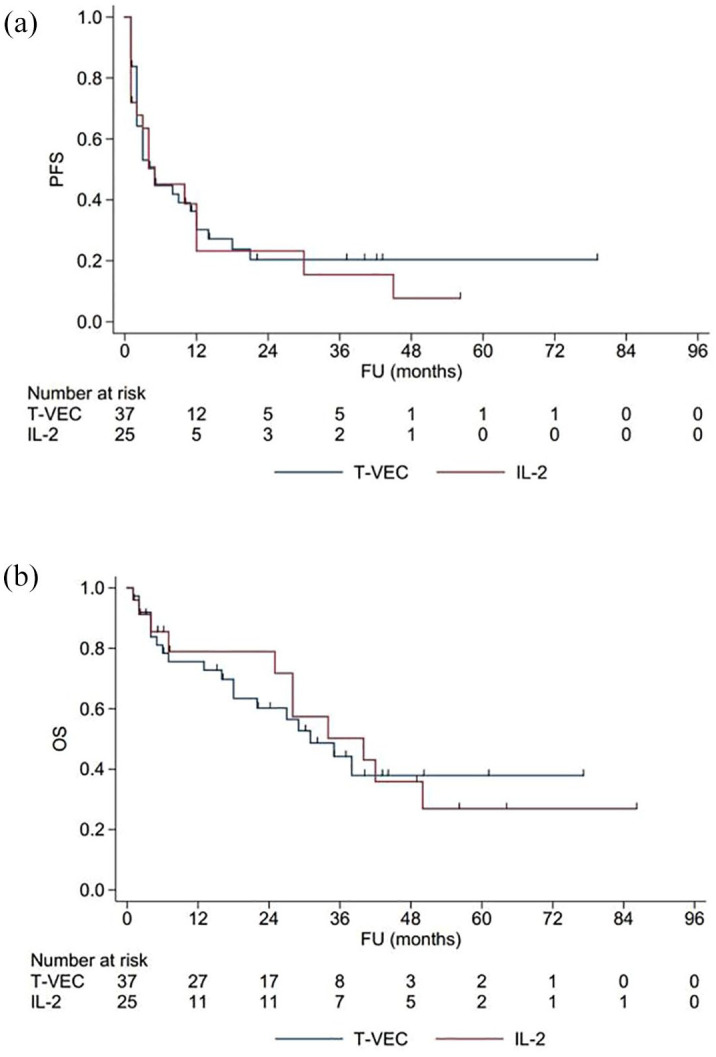
Survival analyses. (a) PFS of patients treated with T-VEC (blue) and IL-2 (red). PFS was defined as the time between the start of the intralesional treatment and entry in stage IV (distant metastases), locoregional progression, or death. (b) The OS of patients. OS was defined as the time between the first intralesional treatment and death for all subgroups. *p*-Values of the log-rank test in PFS *p* = 0.790 and OS *p* = 0.894 indicate no statistical significance between the subgroups. FU, follow-up; IL-2, interleukin 2; OS, overall survival; PFS, progression-free survival; T-VEC, talimogene laherparepvec.

The median PFS of the whole cohort was 5.0 months (95% confidence interval (CI), 0.01–11.80). No significant difference in PFS was observed among the two subgroups in the log-rank test (*p* = 0.790). In both subgroups, the median PFS was 5 months (Figure 2 and Table 2). The median OS of the whole cohort was 34.0 months (95% CI, 23.25–44.65). No significant OS could be detected between the subgroups of T-VEC and IL-2 (*p* = 0.894; Figure 2 and Table 2). The longest median OS was observed in the IL-2 subgroup with 40 months (95% CI, 18.76–61.24), followed by the subgroup with T-VEC with 31 months (95% CI, 12.65–49.35). Nearly half of the cohort (45.2%) experienced a progression to stage IV. The percentage of patients who transitioned to stage IV did not differ significantly between the two subgroups.

The best overall response rate (ORR) of the intralesional treated metastases was 37.1% (*n* = 23) for the entire cohort. In the T-VEC subgroup, the ORR was 32.4% (*N* = 12), while in the IL-2 subgroup, it was 44.0% (*n* = 11). No significant difference was observed between the two subgroups (*p* = 0.355). Overall, nine patients (24.3%) achieved a complete response (CR) in the T-VEC group, and six patients (24.0%) achieved CR in the IL-2 group. The rate of progressive disease (PD) was also similar between the two groups, at approximately 45% (Table 2). No significant difference was observed in the ORR of intralesional therapy between patients who received prior adjuvant systemic therapy and those who did not, for both T-VEC (*p* = 0.774) and IL-2 (*p* = 0.670).

Ten patients received both intralesional therapies during the course of their disease. The best response rates for these patients are shown in detail in Supplemental Table 1. Among the seven patients who received T-VEC first, followed by IL-2, three patients (42.9%) achieved a CR, and two patients (28.6%) had stable disease (SD). One patient experienced PD, and one is still undergoing treatment, with the response yet to be assessed under the second intralesional therapy. By contrast, all three patients who received T-VEC as a second-line therapy (after IL-2) experienced PD.

### Local side effects at the injection site and fever were the most commonly observed AEs

**Figure 3.**
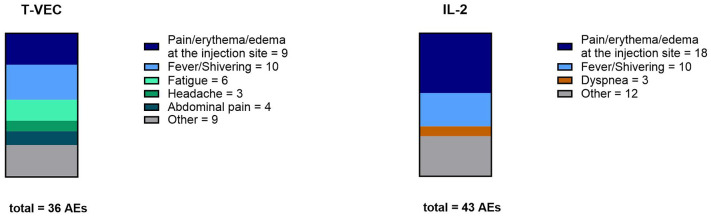
AEs in comparison. AEs with at least three mentions were included in the graphical representation. AEs that were mentioned only 1–2 times were grouped into the category “others” for clarity. AE, adverse events.

In both subgroups, local side effects at the injection site (pain, erythema, and/or edema) and fever/shivering were the most frequently mentioned AEs (Figure 3). Dyspnea (*N* = 3), hypertension (*N* = 1), tachycardia (*N* = 2), lack of appetite (*N* = 2), arthralgia (*N* = 1), and flush (*N* = 1) were reported exclusively in IL-2 patients, whereas headache (*N* = 3), abdominal pain (*N* = 4), diarrhea (*N* = 2), sleep disorder (*N* = 1), and herpes labialis (*N* = 2) were reported exclusively in T-VEC patients. A complete list of all side effects and the number of documented AEs per patient, categorized by the intralesional therapy subgroups, are shown in Supplemental Table 2. A proportion of 48.6% of patients had no AEs in the T-VEC subgroup, whereas the proportion in the IL-2 subgroup was only 16.0%. No patient in the T-VEC group had more than three AEs, whereas in the IL-2 subgroup, 12.0% of patients suffered four AEs. No patient exhibited more than four reported AEs. Severe AEs resulting in discontinuation of intralesional therapy were not observed in this cohort. One patient in the T-VEC subgroup and one patient in the IL-2 subgroup chose to discontinue the therapy due to side effects, regardless of the physician’s recommendation to continue treatment (Supplemental Table 2).

## Discussion

Skin metastases present significant challenges, as they can lead to complications such as ulceration, bleeding, and unpleasant odor, which may negatively impact patients’ quality of life.^1,2^ Intralesional T-VEC and IL-2 injections have both been used in the treatment of melanoma metastases for years, but they differ in many aspects, such as potential AEs, biosafety protocols, and the fact that T-VEC has EMA/FDA approval while intralesional IL-2 does not.^9,12,23^ We aimed to investigate their differences in a real-world cohort of melanoma stage III patients treated with T-VEC and/or IL-2 from January 2016 to September 2024 at the University Hospital Tuebingen.

The most commonly reported side effects of T-VEC in the literature are as follows: fatigue (50.3%), chills (48.6%), fever (42.8%), nausea (35.6%), influenza-like symptoms (30.5%), and injection site pain (27.7%).^24^ Accordingly, we detected these AEs in our patients’ medical records, however to a lower percentage. For IL-2, a meta-analysis revealed that the most commonly reported side effects were pain and swelling, fever, and flu-like symptoms (25%–85%), as well as mild nausea and emesis (40%), consistent with our data.^25^ While two cases of herpes labialis have been reported in our cohort under treatment with T-VEC, IL-2 is not associated with the risk of viral transmission. However, it can cause in rare cases, severe systemic AEs, such as capillary leak syndrome, particularly when applied intravenously, necessitating careful patient monitoring and making it mostly injected in an inpatient setting.^26^ Severe AEs were not observed in our cohort. T-VEC was applied in an outpatient setting, whereas IL-2 was given in an inpatient setting. However, this discrepancy may give a potential bias due to the more consistent documentation practices associated with inpatient care. Therefore, direct comparisons of documented AEs should be interpreted with caution, highlighting the need for further prospective evaluation.

In the T-VEC subgroup, we revealed an ORR of 32.4% higher than the ORR in the OPTiM phase III trial (26.4%) and lower than in other real-world cohorts.^27–29^ However, direct comparison with these cohorts is limited, as the OPTiM trial also included stage IV patients, and approximately 54.8% of patients in our study received concomitant systemic therapy. In the context of survival analysis, the final results of the OPTiM phase III trial confirmed that T-VEC significantly improved the OS compared to the control arm with granulocyte-macrophage colony-stimulating factor (GM-CSF). The median OS for the T-VEC cohort was 23.3 months, compared to 18.9 months for the GM-CSF-treated patients.^27^ However, in the Masterkey-265 study, a phase III study investigating PFS and OS between the subgroups receiving pembrolizumab with T-VEC compared to the subgroup receiving pembrolizumab without T-VEC, no survival advantage was observed for either PFS or OS.^21^ There are no prospective data available for intralesional IL-2 or IL-2 agents in combination with the currently available systemic therapies, that is, ICI regarding PFS and OS. In a small retrospective single-center study of 27 patients, intralesional IL-2 was associated with improved PFS and OS in the subgroup of patients with locoregional progression and no active distant metastases.^20^ Given this data and considering the fact that intralesional treatment of cutaneous and subcutaneous metastases normally would not influence patients’ survival, we decided to focus our retrospective analysis only on melanoma stage III patients. In these patients, treatment is focused also on locally advanced tumor, skin, and subcutaneous metastases. However, we found no statistically significant survival difference between the different intralesional therapy subgroups, neither for PFS nor for OS.

Furthermore, the percentage of patients who experienced a progression into stage IV did not differ significantly between the two subgroups. Regarding our data in relation to the Masterkey-265 data, it could be assumed that neither intralesional therapy has an impact on OS or PFS in combination with ICI. However, the potential psychological impact and improvement in quality of life that the response of skin metastases may have on the patients was not assessed. In fact, the two intralesional therapies may be more supportive, acting as an adjunct to systemic therapy, rather than being a life-prolonging approach. In a small cohort of seven patients, IL-2 demonstrated promising results when used as a second-line therapy after T-VEC. More than 40% of these patients achieved a CR in the second line, and approximately 30% had SD. However, the small sample size limits the ability to conclude the efficacy of IL-2 as second-line therapy following T-VEC. Further studies with larger cohorts are necessary to confirm these preliminary findings.

The reported lower percentage of AEs in our cohort might be attributed to the fact that the available documentation on recorded side effects was taken from the medical records. In all retrospective studies, information obtained from medical records may be incomplete. By contrast, prospective studies focus on specific details in advance; therefore, the risk of underassess important information is reduced. It should also be noted that the small cohort size (*n* = 62) reduces the statistical power of our findings. Furthermore, although no significant differences were observed in the distribution of melanoma stages at the start of intralesional therapy, a potential difference in tumor burden between the two groups cannot be excluded, as the size of cutaneous metastases is not reflected in the AJCC classification.

## Conclusion

While the two intralesional therapies differ in safety profiles and handling, as well as in percentages of AEs, our comparative retrospective analysis demonstrates no significant difference in efficacy. To reach definitive conclusions, additional prospective studies are required. It could also be valuable to compare other local treatments for cutaneous melanoma metastases, such as isolated limb perfusion, electrochemotherapy, and radiotherapy. However, in centers where the complex logistics required for T-VEC cannot be guaranteed, or where there is a risk of viral transmission (e.g., during pregnancy in the immediate environment), the use of T-VEC is not feasible. In this context, IL-2 offers an equal alternative regarding PFS, OS, and best response in this initial retrospective evaluation.

## Supplemental Material

sj-docx-1-tam-10.1177_17588359251324035 – Supplemental material for Comparative real-world outcomes of stage III melanoma patients treated with talimogene laherparepvec or interleukin 2 Supplemental material, sj-docx-1-tam-10.1177_17588359251324035 for Comparative real-world outcomes of stage III melanoma patients treated with talimogene laherparepvec or interleukin 2 by Markus Reitmajer, Lena Nanz, Nina Müller, Ulrike Leiter, Teresa Amaral, Valentin Aebischer, Lukas Flatz and Andrea Forschner in Therapeutic Advances in Medical Oncology

sj-docx-2-tam-10.1177_17588359251324035 – Supplemental material for Comparative real-world outcomes of stage III melanoma patients treated with talimogene laherparepvec or interleukin 2 Supplemental material, sj-docx-2-tam-10.1177_17588359251324035 for Comparative real-world outcomes of stage III melanoma patients treated with talimogene laherparepvec or interleukin 2 by Markus Reitmajer, Lena Nanz, Nina Müller, Ulrike Leiter, Teresa Amaral, Valentin Aebischer, Lukas Flatz and Andrea Forschner in Therapeutic Advances in Medical Oncology

sj-docx-3-tam-10.1177_17588359251324035 – Supplemental material for Comparative real-world outcomes of stage III melanoma patients treated with talimogene laherparepvec or interleukin 2 Supplemental material, sj-docx-3-tam-10.1177_17588359251324035 for Comparative real-world outcomes of stage III melanoma patients treated with talimogene laherparepvec or interleukin 2 by Markus Reitmajer, Lena Nanz, Nina Müller, Ulrike Leiter, Teresa Amaral, Valentin Aebischer, Lukas Flatz and Andrea Forschner in Therapeutic Advances in Medical Oncology

## Declarations

**Ethics approval and consent to participate:** This retrospective analysis was approved by the local Ethics Committee of the University of Tuebingen (project number 040/2024BO2) and followed the general recommendations outlined in the Declaration of Helsinki. All patients included in this registry provided written informed consent for documentation of their clinical data in the central melanoma registry for research purposes and publications.

**Consent for publication:** We confirm that neither the submitted manuscript nor any similar manuscript, in whole or in part, is under consideration, in press, published, or reported elsewhere. All authors had read and approved the final version of the manuscript, its contents, and its submission to the Journal *Therapeutic Advances in Medical Oncology*.

**Availability of data and materials:** The datasets used and/or analyzed during the current study are available from the corresponding author upon reasonable request.
