# Supplementary material for: Comparative real-world outcomes of stage III melanoma patients treated with talimogene laherparepvec or interleukin 2
Source: Ther Adv Med Oncol. 2025 Apr 1;17:17588359251324035. doi: 10.1177/17588359251324035 (PMC11960150; doi:10.1177/17588359251324035)
Supplement: sj-docx-2-tam-10.1177_17588359251324035 – Supplemental material for Comparative real-world outcomes of stage III melanoma patients treated with talimogene laherparepvec or interleukin 2 [file sj-docx-2-tam-10.1177_17588359251324035.docx]

## Supplementary Table 1 Details of patients treated with both applied intralesional therapies

Following abbreviations were used: number of patients (N), complete response (CR), partial response (PR), stable disease (SD), progressive disease (PD) and overall response rate (ORR), months (mo.), no data available (n/a)

|  | N | % | - | - | - |
| --- | --- | --- | --- | --- | --- |
| **Treatment with both intralesional therapies** | 10 | 100 | - | - | - |
| First T-VEC, followed by IL2 | 7 | 70 | - | - | - |
| First IL-2, followed by T-VEC | 3 | 30 | - | - | - |
| **Local best response first intralesional therapy** | **N** | **%** | **Local best response second intralesional therapy** | **N** | **%** |
| **First T-VEC** | 7 | 100 | **Followed by IL** | 7 | 100 |
| CR | 1 | 14.3 | CR | 3 | 42.9 |
| PR | 0 | 0 | PR | 0 | 0 |
| SD | 3 | 42.9 | SD | 2 | 28.6 |
| PD | 3 | 42.9 | PD | 1 | 14.3 |
| n/a. | 0 | 0 | n/a. | 1 | 14.3 |
| **First IL-2** | 3 | 100 | **Followed by T-VEC** | 3 | 100 |
| CR | 0 | 0 | CR | 0 | 0 |
| PR | 1 | 33.3 | PR | 0 | 0 |
| SD | 0 | 0 | SD | 0 | 0 |
| PD | 2 | 66.7 | PD | 3 | 100 |
