# Supplementary material for: Comparative real-world outcomes of stage III melanoma patients treated with talimogene laherparepvec or interleukin 2
Source: Ther Adv Med Oncol. 2025 Apr 1;17:17588359251324035. doi: 10.1177/17588359251324035 (PMC11960150; doi:10.1177/17588359251324035)
Supplement: sj-docx-3-tam-10.1177_17588359251324035 – Supplemental material for Comparative real-world outcomes of stage III melanoma patients treated with talimogene laherparepvec or interleukin 2 [file sj-docx-3-tam-10.1177_17588359251324035.docx]

## Supplementary Table 2 Details about adverse events associated with the applied intralesional therapies

Following abbreviations were used: Chi-square test (^C^), Fisher’s exact Test (^F^), p-value (p), number of patients (N)

|  | **T-VEC** | | **Il-2** | | **p^C/F/LR^** |
| --- | --- | --- | --- | --- | --- |
|  |  | |  | |  |
|  | N | % | N | % |  |
| **Stopped therapy due to adverse event(s)** | 1 | 2.7 | 1 | 4.0 | 1.000^F^ |
| Physician decision | 0 | 0 | 0 | 0 |  |
| Patient decision | 1 | 2.7 | 1 | 4.0 |  |
| **Number of documented adverse event(s)**  **per patient** |  |  |  |  | **0.025^C^** |
| zero | 18 | 48.6 | 4 | 16.0 |  |
| one | 5 | 13.5 | 9 | 36.0 |  |
| two | 6 | 16.2 | 5 | 20.0 |  |
| three | 8 | 21.6 | 4 | 16.0 |  |
| four | 0 | 0 | 3 | 12.0 |  |
| **Type of adverse event** |  |  |  |  |  |
| Pain/erythema/edema at the injection site | 9 | 24.3 | 18 | 72.0 |  |
| Fever/chills | 10 | 27.8 | 10 | 40.0 |  |
| Dyspnea | 0 | 0 | 3 | 12.0 |  |
| Hypertension | 0 | 0 | 1 | 4.0 |  |
| Tachycardia | 0 | 0 | 2 | 8.0 |  |
| Nausea | 2 | 5.6 | 2 | 8.0 |  |
| Fatigue | 6 | 16.7 | 1 | 4.0 |  |
| Arthralgia | 0 | 0 | 1 | 4.0 |  |
| Myalgia | 1 | 2.8 | 1 | 4.0 |  |
| Pruritus | 1 | 2.8 | 1 | 4.0 |  |
| Flush | 0 | 0 | 1 | 4.0 |  |
| Lack of appetite | 0 | 0 | 2 | 8.0 |  |
| Herpes labialis | 2 | 5.6 | 0 | 0 |  |
| Sleep disorder | 1 | 2.8 | 0 | 0 |  |
| Headache | 3 | 8.3 | 0 | 0 |  |
| Abdominal pain | 4 | 11.1 | 0 | 0 |  |
| Diarrhea | 2 | 5.6 | 0 | 0 |  |
